# Supplementary material for: GATA transcription factors, SOX17 and TFAP2C, drive the human germ-cell specification program
Source: Life Sci Alliance. 2021 Feb 19;4(5):e202000974. doi: 10.26508/lsa.202000974 (PMC7918644; doi:10.26508/lsa.202000974)
Supplement: Supplementary file 1 [file LSA-2020-00974_SdataFS1_FS4.pdf]

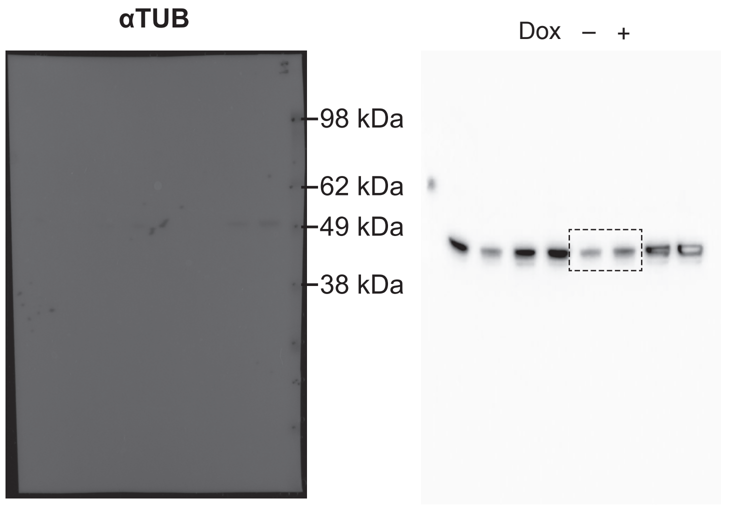

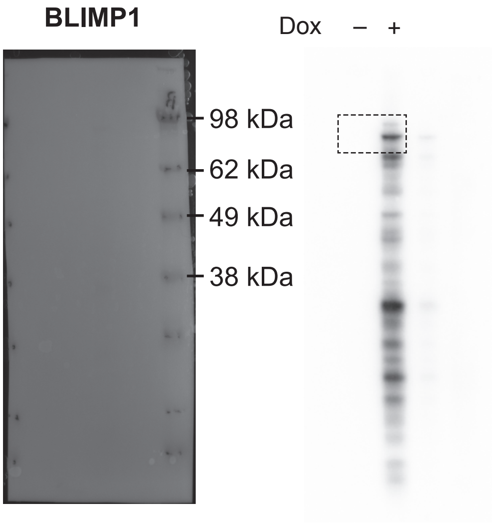

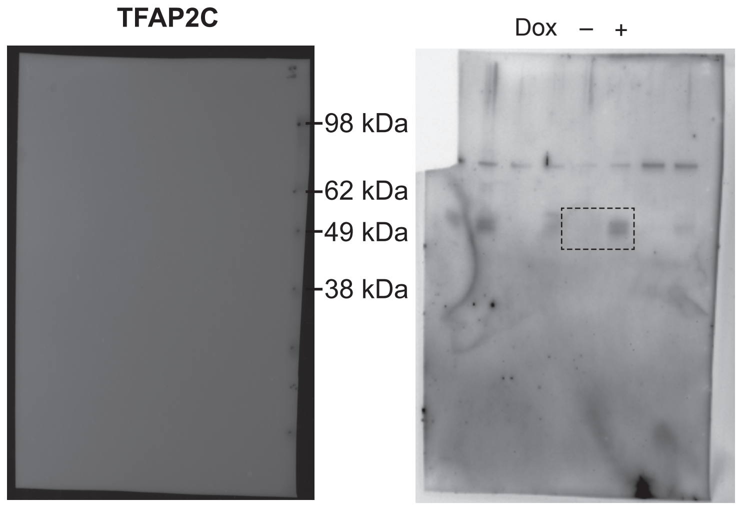

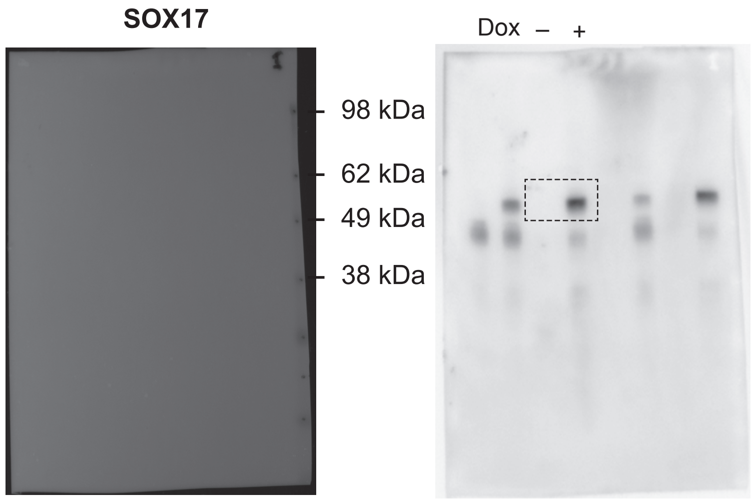

Source images for Figure S1B
Original photographs of the full membranes of the Western blottings for α-TUBULIN, TFAP2C, SOX17 and BLIMP1 in the SOX17/TFAP2C/BLIMP1 clone. The areas used for Figure S1B are indicated by boxes with dotted lines.

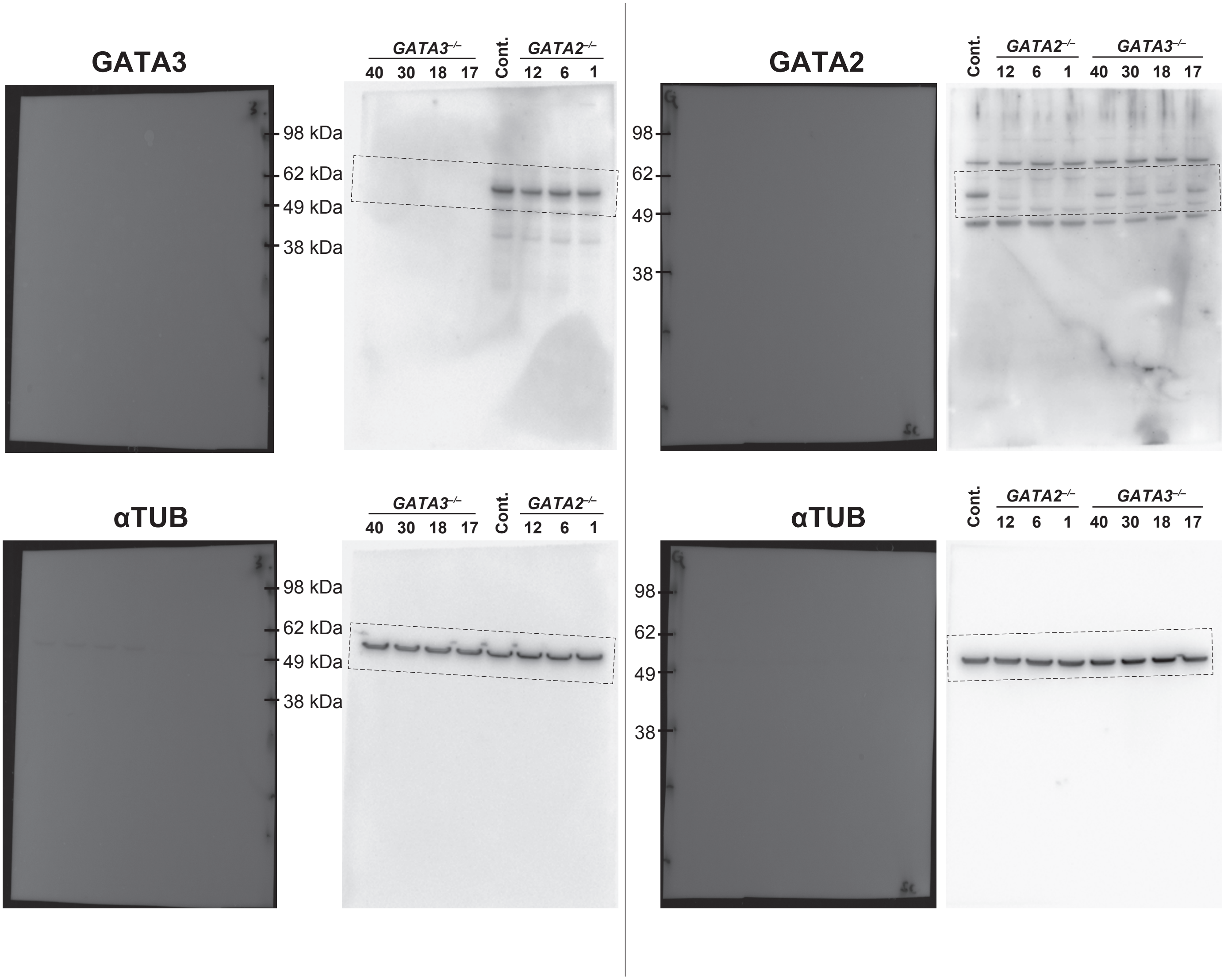

Source images for Figure S4B
Original photographs of the full membranes of the Western blottings for GATA3, GATA2 and α-TUBULIN in the GATA3$^{-/-}$ and GATA2$^{-/-}$ clones. The areas used for Figure S4B are indicated by boxes with dotted lines.

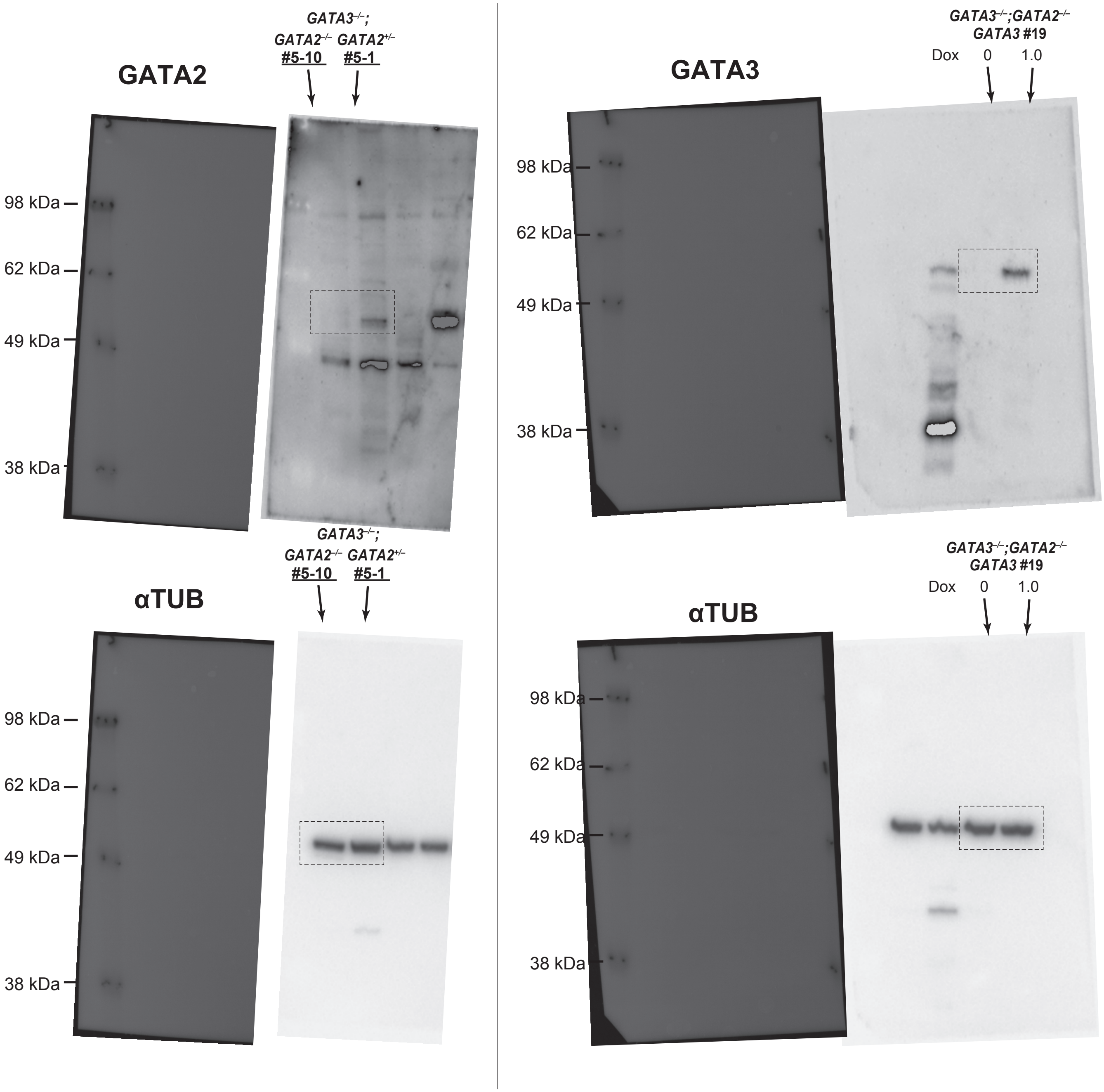

Source images for Figure S4B

Original photographs of the full membranes of the Western blotting for GATA2, GATA3 and α-TUBULIN in GATA3$^{-/-}$;GATA2$^{+/-}$ #5-1, GATA3$^{-/-}$;GATA2$^{-/-}$ #5-10 and GATA3$^{-/-}$;GATA2$^{-/-}$;GATA3 #19 clones. The areas used for Figure S4B are indicated by boxes with dotted lines.
